# Modeling antibody recognition across SARS-CoV-2 and SARS-CoV-1 using epitope-informed transfer learning

**DOI:** 10.3389/fimmu.2026.1886934

**Published:** 2026-08-05

**Authors:** Jimmy B. Yuan, Ryan C. Bruneau, Stephen Won, Aidan Elliot Heller, Thomas Sheffield, Kenneth L. Sale, Brooke Harmon, Le Thanh Mai Pham

**Affiliations:** 1 Biotechnology and Bioengineering, Sandia National Laboratories, Livermore, CA, United States; 2 Bioresource and Environmental Security, Sandia National Laboratories, Livermore, CA, United States; 3 Biosecurity and Bioassurance, Sandia National Laboratories, Livermore, CA, United States

**Keywords:** antibody prioritization, antibody: antigen interaction, computational immunology, epitope modeling, Sarbecovirus, SARS-CoV-1, SARS-CoV-2, transfer learning

## Abstract

Antiviral antibody discovery is constrained by the enormous combinatorial space of antibody paratopes and viral epitopes, while novel approaches to search for these vast possibilities such as machine learning models for antibody affinity prediction typically require large target-specific datasets that are rarely available for emerging pathogens. To address this problem, we investigated the ability of transfer learning to reduce the required training data and still create a robust predictive model of antibody affinities. We show that antibody-affinity prediction can be locally transferred from a data-rich viral target to a related data-limited viral target by combining transfer learning, epitope-centered antigen encoding, antibody CDR descriptors, and targeted experimental validation. Using SARS-CoV-2 receptor-binding-domain (RBD) interaction data as the source domain, we developed dual-encoder neural-network models that capture functional determinants of antibody recognition and supports prediction of SARS-CoV-1 variant:antibody affinities despite limited direct training data. High-throughput ELISA measurements generated compact validation datasets spanning heavy-chain-only antibodies (HCAbs) and conventional antibodies, enabling iterative model refinement through similarity-guided, model-informed data expansion. Across expanded withheld-antibody evaluations, the final models showed improved but antibody-dependent predictive performance, with mean Pearson correlations of 0.71 ± 0.12 for SARS-CoV-2 and 0.63 ± 0.09 for SARS-CoV-1, and best-performing holdouts reaching 0.85 and 0.78, respectively. These results demonstrate that existing immunological datasets can support feature-constrained transfer learning for data-efficient prioritization of antibody:antigen interactions across closely related Sarbecoviruses, particularly when conserved epitope regions can be aligned and limited target-specific measurements are available for calibration.

## 1 Introduction

Antibody discovery against viral pathogens is challenging due to the enormous combinatorial space of both antibody paratopes (1) and antigen epitopes (2, 3). Even for a single viral antigen, the theoretical antibody sequence space is vast. For example, a 40-residue region can in principle adopt 20^40^ ≈ 10^52^ distinct amino acid sequences, and although only a small fraction of this space is expected to be structurally viable, developable, or antigen-binding, the functional diversity space surpasses the feasibility to experimentally map and understand all possible interactions with other proteins of interest such as host receptors or antibodies. Experimental technologies such as phage display, deep mutational scanning (DMS), and high-throughput enzyme-linked immunosorbent assays (ELISA) have accelerated functional antibody identification, but they remain costly and time-consuming, especially during rapid-response scenarios for emerging viruses, such as SARS-CoV-2 variant surveillance (4, 5). Consequently, there is a pressing need for computational strategies capable of inferring antibody:antigen interactions without requiring exhaustive laboratory screening to improve quick deployment of medical countermeasures to viral threats.

Predictive machine learning (ML) techniques provide a powerful framework for addressing this challenge: supervised learning algorithms learn patterns from vast datasets via cycles of gradient descent or other optimization algorithms to create a model directed towards predicting a defined target variable, which are increasingly used in diverse scientific fields including biology (6). Such models trained on experimentally labeled antigen:antibody interaction datasets have emerged as promising tools for predicting binding, mutational effects, and antibody escape across incompletely sampled sequence landscapes. For example, deep-learning studies have shown that antibody sequence information can be used to identify antigen-specific variants and guide optimization, highlighting the value of supervised learning for sequence-to-function prediction (7). Yet these methods typically require large, diverse, and well-curated training datasets, which are not available for newly emerging or poorly characterized pathogens. In addition, recent analyses indicate that currently available antibody:antigen affinity datasets are still too limited in both size and diversity to support consistently robust generalization. As antigenic drift and viral evolution push new variants outside the training distribution, models trained on a single viral species may therefore lose predictive accuracy and become biased toward dataset-specific features rather than transferable interaction rules (8).

Transfer learning, in which a model trained on one task is adapted to a related task, offers a practical strategy for overcoming limited generalizability in data-sparse settings. Because related viruses often share conserved sequence, structural, and functional features, information learned from antibody binding to one viral antigen may remain informative for predicting binding to an antigen from a related virus. Although transfer learning has shown promise for predicting immune-relevant viral properties, its application to quantitative antigen:antibody affinity prediction remains relatively underexplored.

**Figure 1:**
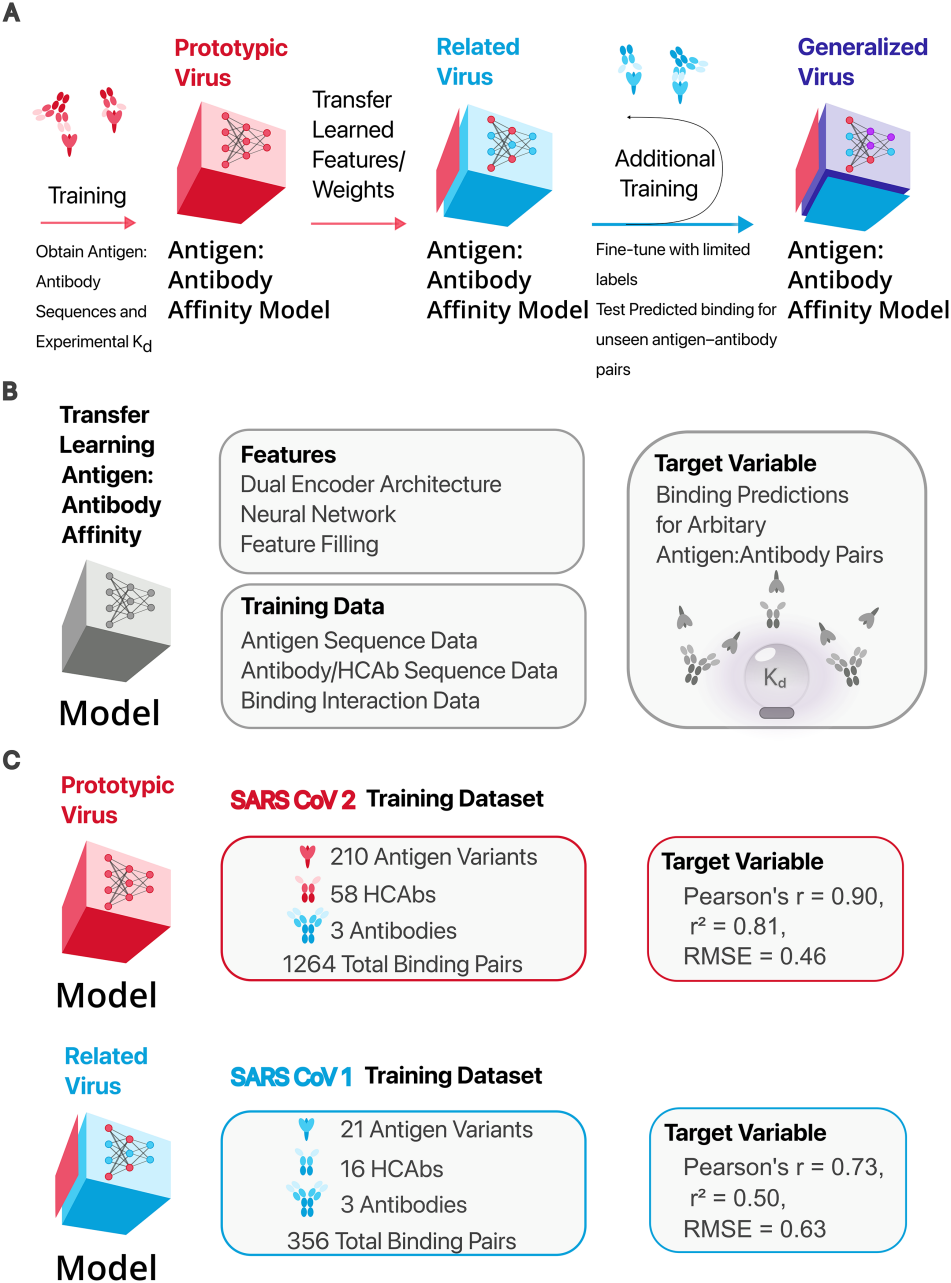
Feature-constrained transfer-learning framework for antigen: antibody affinity prediction across related viruses. **(A)** A source model trained on a data-rich virus is transferred to a related, data-limited virus and refined with limited target-virus binding measurements. **(B)** The framework uses aligned epitope-patch features, antibody CDR descriptors, and experimentally derived relative affinity values to prioritize antigen: antibody pairs. Model performance is evaluated using Pearson correlation, R², and RMSE under withheld-antibody validation. **(C)** Summary of the SARS-CoV-2 source datasets and SARS-CoV-1 target datasets used for model training, transfer, and refinement.

Here, we address this gap by extending antigen:antibody affinity prediction from data-rich viruses to related, data-limited viruses using only small experimentally derived datasets. Using a framework originally developed for SARS-CoV-2 RBD:antibody interactions, we generated affinity predictions for SARS-CoV-1 RBD variants against a panel of conventional antibodies and HCAbs despite limited direct measurements. To evaluate this approach, we constructed a compact, high-quality experimental dataset using titration ELISAs across a limited set of antigen variants and antibodies. This dataset provides an experimental benchmark for cross-virus affinity prediction and shows that transfer learning can yield biologically grounded predictions of antibody recognition (**Figure 1**). Our results support a practical, data-efficient framework for reusing existing immunological datasets to predict antibody:antigen interactions across related, data-limited viruses in the presented case, between closely related viruses in the Sarbecovirus subgenus. By enabling accurate affinity prediction with minimal additional measurements, this approach can reduce dependence on large-scale screening, accelerate early therapeutic prioritization, and may aid in creating more generalized predictive models at the viral genus level.

## 2 Results

Because very limited experimental affinity data existed for SARS-CoV-1 RBD:antibody interactions, we developed a cross-virus predictive framework that leverages a richer SARS-CoV-2 interaction dataset to inform SARS-CoV-1 modeling. Our strategy was to build a high-performance neural network model to predict SARS-CoV-2 variants:HCAb/conventional antibody interaction affinities (see Methods) to learn representations of both antibody sequences and SARS-CoV-2 RBD epitope sequences. Then after targeted data expansion and epitope mapping, the learned representations were transferred to a model predicting SARS-CoV-1 variant:HCAb/conventional antibody interaction affinities. By limiting viral input features to conserved neutralizing epitope patches and representing antibodies through physicochemical CDR descriptors, the models emphasized functional binding determinants while reducing noise from full-length sequence representations. **Table 1** summarizes the ML models developed and evaluated in this study, including their data sources, predictive targets, model architectures, and validation strategies. All modeling datasets are provided in **Supplementary File S1** of the **Supplementary Information**. We progressively improved prediction of relative SARS-CoV-1 antigen:antibody affinity by integrating three complementary strategies: (1) epitope-informed transfer learning from SARS-CoV-2 antigen:antibody affinity models, (2) sequence-module prioritization within a dual-encoder neural-network architecture, and (3) similarity-guided dataset enrichment using underrepresented antibodies, SARS-CoV-2 bridge variants, and experimentally measured SARS-CoV-1 antigen:antibody pairs.

**Table 1:** Summary of ML models for SARS-CoV-2:antibody and SARS-CoV-1:antibody affinity prediction.

Stage	Model comparison	Data used	Model/feature change	Validation	Performance summary
Baseline SARS-CoV-2 model	S2.0_NN	D2.1	Fully connected NN using full-length antigen features and HCAb CDR descriptors	80/20 random split	S2: Pearson’s r = 0.85, R² = 0.72, RMSE = 0.64
Epitope-feature model	S2.1a_NN → S1.1a_NN	D2.1 + D1.1	Transfer-learning NN using BLOSUM62-encoded epitope patches and HCAb CDR descriptors	LOAO/SARS-CoV-1 transfer evaluation	S2: Pearson’s r = 0.73, R² = 0.50, RMSE = 0.60; S1: Pearson’s r = 0.47, R² = 0.17, RMSE = 1.01
Dual-encoder model	S2.1b_NN → S1.1b_NN	D2.1 + D1.1	Separate antibody and antigen encoder branches	LOAO/SARS-CoV-1 transfer evaluation	S2: Pearson’s r = 0.78, R² = 0.57, RMSE = 0.58; S1: Pearson’s r = 0.55, R² = 0.14, RMSE = 1.03
Similarity-guided expansion, round 1	S2.2_NN → S1.2_NN	D2.2 = D2.1 ∪ AL2.1; D1.1	Updated SARS-CoV-2 source model with similarity-guided data expansion	LOAO/transfer evaluation	S2: Pearson’s r = 0.75, R² = 0.53, RMSE = 0.62; S1: Pearson’s r = 0.56, R² = 0.13, RMSE = 1.03
Similarity-guided expansion, round 2	S2.3_NN → S1.3_NN	D2.2; D1.2 = D1.1 ∪ AL1.1	Added SARS-CoV-1 experimental calibration data	LOAO/transfer evaluation	S2: Pearson’s r = 0.67, R² = 0.09, RMSE = 0.85; S1: Pearson’s r = 0.68, R² = 0.36, RMSE = 0.67
Final HCAb + conventional antibody model	S2.4_NN → S1.4_NN	D2.2 + D1.2 + conventional antibody data	Dual-encoder NN with antibody-class indicator	Combined withheld-antibody validation; one HCAb and one conventional antibody withheld	Expanded holdout mean ± SD: S2: Pearson’s r = 0.71 ± 0.12, R² = 0.42 ± 0.19, RMSE = 0.73 ± 0.13; S1: Pearson’s r = 0.63 ± 0.09, R² = 0.35 ± 0.14, RMSE = 0.68 ± 0.08.

*Denotation: S2, SARS-CoV-2 source-model series; S1, SARS-CoV-1 transfer-model series; HCAb, heavy-chain-only antibody; RBD, receptor-binding domain; CDR, complementarity-determining region; LOAO, leave-one-antibody-out; AATopo, amino acid topological descriptors; SOCN, sequence-order-coupling numbers; AL, heuristic model-guided experimental expansion dataset selected using antibody feature-space coverage and antigen sequence/feature similarity rather than formal uncertainty-based acquisition functions.

Models, data sources, architectures, input features, training strategy, and performance metrics (Pearson’s r, R², RMSE) are shown. SARS-CoV-1 models use transfer learning from SARS-CoV-2 to enable accurate prediction with limited data.

### 2.1 Epitope-focused modeling of SARS-CoV-2 variant:antibody affinities using conserved RBD patches

To define influential residues and coherent epitope patches within the SARS-CoV-2 RBD, we integrated published single-mutation effect data with structural, conformational, and epistatic analyses. Site-level importance scores were computed from normalized positive and negative mutation effects and supplemented with weighting for ACE2-contact and Omicron-enriched residues (9) As performed in Sheffield et al., contiguous high-scoring positions were grouped into candidate regions, after which higher-order interactions were quantified using a linear model applied to residuals from the ACE2-binding epistasis framework. The RBD was divided into seven structure-based regions, generating 28 possible region:region interaction pairs whose contributions were evaluated using *t*-statistics; pairs exceeding ±2 standard errors were taken as evidence of cooperative effects. To refine epitope boundaries, we incorporated two complementary sources of information: (1) SEMA 2.0 conformational epitope predictions (10) and (2) interaction-based epitope mapping derived from crystallographic SARS-CoV-2 RBD complexes, including ACE2-bound and antibody-bound structures (PDB: 6LZG, 2AJF, 2DD8, 7R6W). Residues involved in recurrent intermolecular contacts across these complexes, or contributing putative stabilizing interactions defined by hydrogen bonds, salt bridges, or hydrophobic contacts within standard distance cutoffs, were designated as structural epitope sites.

The final epitope region used was obtained by taking the union of residues included in either epitope set. Epitope patches were then concatenated such that no two patches were separated by fewer than two amino acids, yielding a total of ten spatially coherent functional motifs. To extend this annotation to SARS-CoV-1, we aligned the SARS-CoV-1 and SARS-CoV-2 RBDs using MUSCLE v5.1 (11) and mapped epitope residues by identifying their aligned counterparts (**Table 2**). Integrating site importance, region: region interactions, conformational and structural epitope data, and cross-virus alignment yielded a unified set of conserved neutralizing epitope patches that supported restricted-feature model training, comparison to full-RBD models, and prioritization of antibody:antigen variants for downstream experimental validation.

**Table 2:** Cross-virus epitope patch mapping between SARS-CoV-2 and SARS-CoV-1.

Epitope patch ID	SARS-CoV-2	SARS-CoV-1
1	NLCPFGE	NLCPFGE
2	NATRF	NATKF
3	YAWNRKR	YAWERKK
4	VADYSVLYNSASFSTFKC	VADYSVLYNSTFFSTFKC
5	YADSFVIRGDEVRQIAPGQTGKIADY	YADSFVVKGDDVRQIAPGQTGVIADY
6	FTGCVIAWNSNNLDSKV	FMGCVLAWNTRNIDATS
7	NYNYLYRLFRKSNLKPFERDI	NYNYKYRYLRHGKLRPFERDI
8	AGSTP	PDGKP
9	GVEGFNCYFPL	PPALNCYWPL
10	PTNGVGYQPY	TTGIGYQPY

Each epitope patch ID denotes a structurally or sequence-defined epitope region identified on the viral antigen.

As a starting point, we leveraged the previously published SARS-CoV-2:HCAb interaction dataset from Sheffield et al. (D2.1), comprising 1,254 SARS-CoV-2 RBD variant:HCAb pairs across 59 HCAbs and 201 RBD variants. While the original random forest framework achieved strong predictive performance within SARS-CoV-2, it was not readily transferable to SARS-CoV-1. We therefore reformulated the prediction task using a fully connected neural network, which enabled transfer learning from the data-rich SARS-CoV-2 setting to the smaller SARS-CoV-1 affinity datasets generated in this study.

To establish a baseline for antibody:antigen affinity prediction, we first trained a fully connected neural network, S2.0_NN, on the SARS-CoV-2 RBD variant:HCAb dataset from Sheffield et al. (9). This model achieved strong overall performance (Pearson’s r = 0.85, R² = 0.72, RMSE = 0.64), demonstrating that mutational effects on RBD binding could be learned effectively from paired variant:antibody data. However, because full-sequence representations are poorly suited for transfer across related viral systems, we next reformulated the problem around conserved functional interfaces. We therefore constructed an epitope-centered framework in which antigen inputs were represented as epitope-restricted RBD patches (**Table 2**) and antibody inputs as CDR-focused HCAb descriptors, producing model S2.1a_NN.

**Figure 2:**
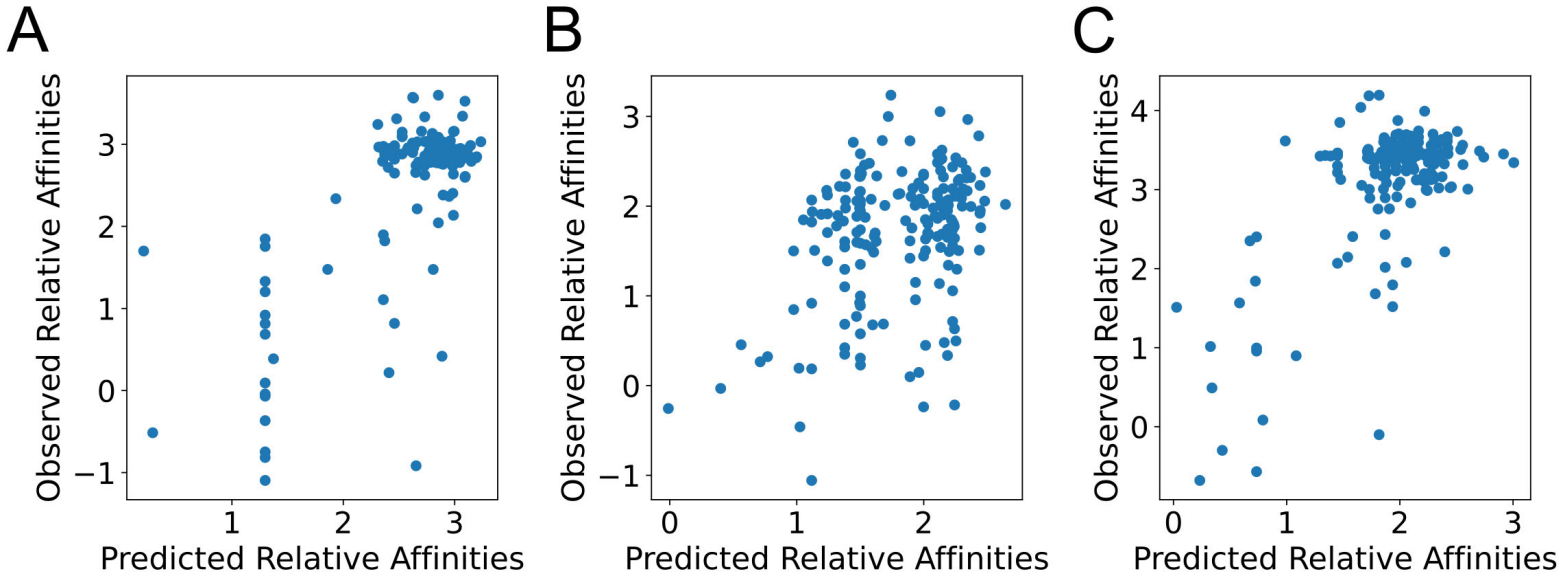
LOAO validation of the SARS-CoV-2 HCAb affinity model. For each analysis, all variant:antibody pairs corresponding to one HCAb were excluded from training and used only for testing, allowing assessment of model generalization to an unseen antibody. Performance was strongest when KP1C9 **(A)** was withheld (Pearson’s r = 0.73, R² = 0.50, RMSE = 0.63), weaker for BP3D5 **(B)** (Pearson’s r = 0.36, R² = 0.05, RMSE = 0.73) and poorest for BP2A6 **(C)** (Pearson’s r = 0.64, R² = −1.69, RMSE = 1.36).

We reasoned that, if successful, such a representation should improve transferability across antibodies by emphasizing interaction-relevant features rather than global sequence context. To test this directly, we performed leave-one-antibody-out (LOAO) analyses in which all examples associated with a given HCAb were withheld during training and reserved for testing. To select antibodies for LOAO evaluation, we first considered the number of associated SARS-CoV-2 variant measurements for each antibody, reasoning that withheld antibodies should provide sufficiently large test sets for meaningful assessment. BP2A6, BP3D5, and KP1C9 were prioritized because they had the largest variant coverage in the dataset (**Supplementary Information**, **Supplementary Figure 1**). The model performed best when KP1C9 was withheld (Pearson’s r = 0.73, R² = 0.50, RMSE = 0.63), consistent with this antibody occupying a region of feature space that was reasonably captured by the remaining training examples. In contrast, predictive performance dropped for BP3D5 (Pearson’s r = 0.36, R² = 0.05, RMSE = 0.73) and deteriorated further for BP2A6 (Pearson’s r = 0.64, R² = −1.69, RMSE = 1.36), indicating more limited extrapolation to antibodies with less well-represented binding determinants (**Figure 2**). Subsequent analysis of CDR feature distributions suggested that KP1C9 was better represented within the remaining antibody feature space than BP2A6 or BP3D5, consistent with its stronger LOAO performance (**Supplementary Information**, **Supplementary Figure 2**).

To better capture the relational structure of antibody:antigen recognition, we next replaced the single-branch network with a dual-encoder neural network architecture comprising separate encoding pathways for antigen and antibody features. This yielded model S2.1b_NN and improved predictive performance for the KP1C9 holdout (Pearson’s r = 0.78, R² = 0.57, RMSE = 0.58). These results suggest that epitope-centered antigen representations help transfer modeling across related viral RBDs, and that separating antigen and antibody encodings improves generalization to unseen binders.

### 2.2 Transfer of SARS-CoV-2 antibody–variant affinity information improves modeling of SARS-CoV-1 variant:antibody interactions

Information learned from modeling SARS-CoV-2 variant:antibody affinities can be leveraged to support prediction of SARS-CoV-1 variant:antibody affinities. To test this, we developed a model for predicting relative affinity, $a_{R}^{\text{ab}:\text{var}}$ for SARS-CoV-1 RBD variant: antibody interactions. The initial SARS-CoV-1 model, S1.1a_NN, used the same fully connected architecture and hyperparameter settings as the SARS-CoV-2 epitope-based model S2.1a_NN. To enable transfer learning, the parameters of the first two layers of S1.1a_NN were initialized from the trained weights of S2.1a_NN and frozen during backpropagation, thereby preserving shared epitope-level representations learned from the SARS-CoV-2 dataset.

**Figure 3:**
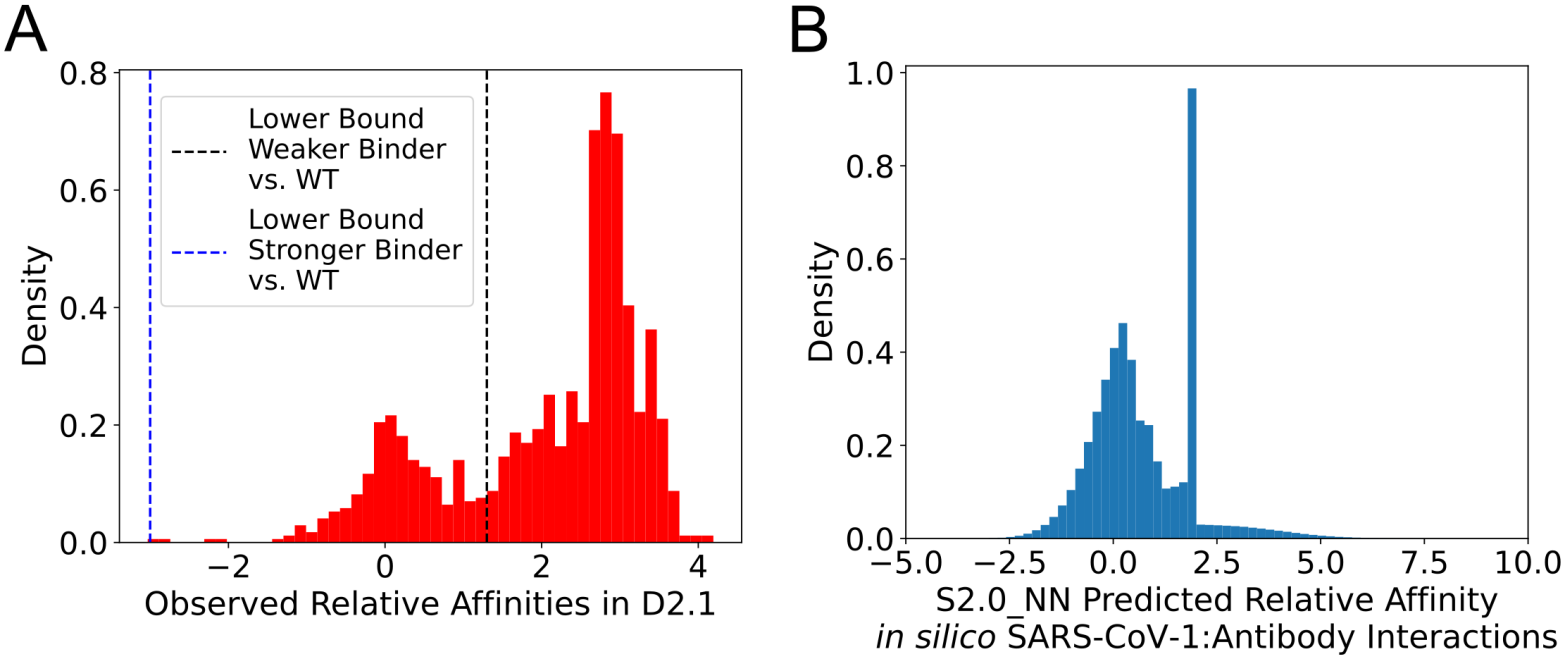
Comparison of affinity-value distributions between in-house SARS-CoV-2 data and in silico SARS-CoV-1 predictions. **(A)** Histogram of measured in-house SARS-CoV-2 HCAb : RBD relative affinity data. **(B)** Histogram of in silico predicted SARS-CoV-1 HCAb : RBD relative affinity values. The distinct distributions indicate a substantial shift between the SARS-CoV-2 and SARS-CoV-1 modeling spaces.

To construct the initial SARS-CoV-1 prediction space, SARS-CoV-1 RBD variants were representing using amino acid substitutions observed across the SARS-CoV-2 RBD mutational landscape, and predictions were made using a subset of the same HCAbs included in the SARS-CoV-2 interaction dataset. Under this pre-transfer configuration, the initial epitope-based transfer model was able to predict relative affinity values for SARS-CoV-1 RBD variant:HCAb interactions. These results established a baseline framework for SARS-CoV-1 affinity prediction before experimental SARS-CoV-1 calibration and transfer-learning refinement. Comparison of the in-house SARS-CoV-2 affinity distribution with the *in-silico* SARS-CoV-1 prediction distribution revealed a marked distributional shift between source and target spaces (**Figure 3**), highlighting the challenge of direct cross-virus prediction and motivating subsequent target-domain data collection and model refinement.

**Figure 4:**
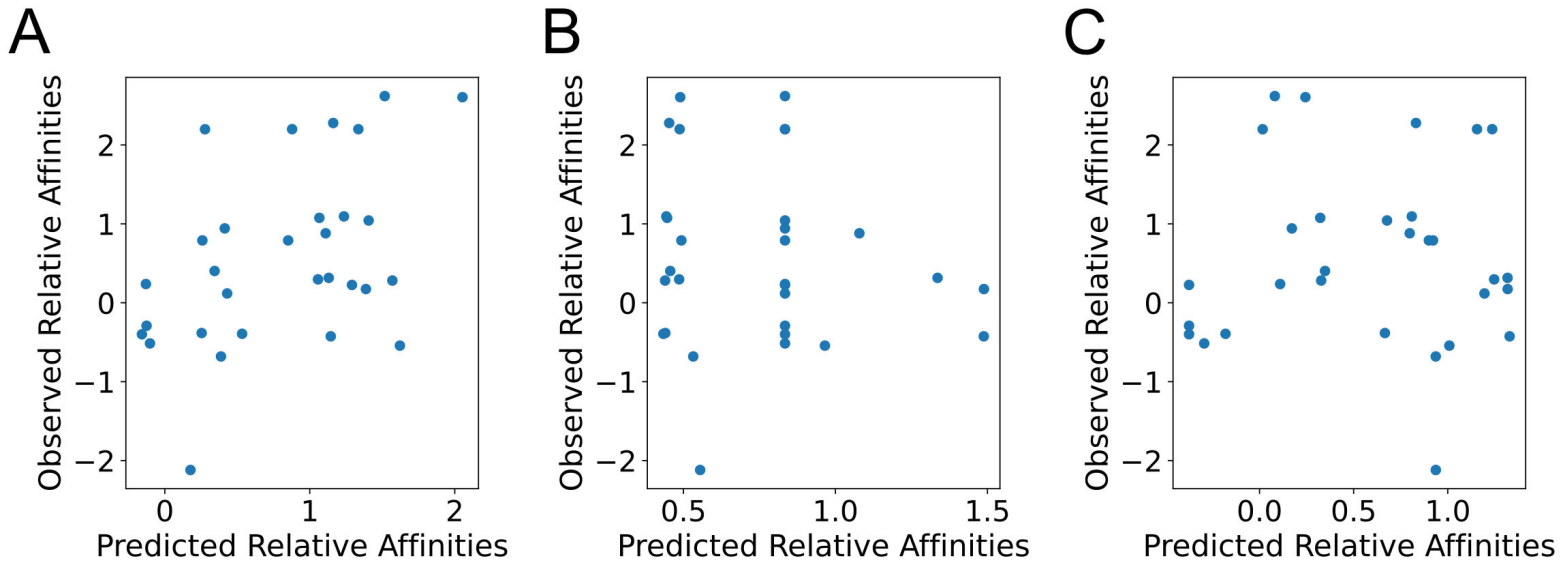
Leave-one-antibody-out (LOAO) validation on SARS-CoV-1 HCAbs. Scatter plots show predicted versus observed relative affinities (Methods) for SARS-CoV-1 RBD variant:HCAb interactions when individual antibodies were withheld from training: KP1C9 **(A)**, BP3D5 **(B)** and BP2A6 **(C)**. Model performance varied substantially across antibodies. Predictive generalization was strongest for KP1C9 (Pearson r = 0.47, R² = 0.17, RMSE = 1.01), whereas performance was minimal for BP2A6 (Pearson r = 0.02, R² = −0.24, RMSE = 1.23) and BP3D5 (Pearson r = −0.14, R² = −0.17, RMSE = 1.20), consistent with antibody-specific gaps in training-set coverage.

We next used this baseline transfer-learning model to prioritize SARS-CoV-1 RBD variants for experimental validation using in-house produced HCAbs. Based on model predictions, 23 SARS-CoV-1 RBD variants were selected for testing across a subset of HCAbs, yielding 250 usable variant–antibody affinity measurements after excluding pairs with unreliable curve fits or insufficient binding signal. Evaluation of this experimentally calibrated epitope-based model provided a baseline for cross-virus affinity prediction and revealed antibody-dependent generalization under LOAO validation (**Figure 4**). As observed for the SARS-CoV-2 model S2.1a_NN, leave-one-antibody-out analysis of S1.1a_NN revealed substantial antibody-dependent variability in generalization. The model retained modest predictive power for KP1C9 (Pearson’s r = 0.47, R² = 0.17, RMSE = 1.01), whereas predictive transfer was minimal for BP2A6 and BP3D5, for which correlations were near zero or negative (**Figure 4**). These results indicate that SARS-CoV-2-derived representations provide a useful initialization for cross-virus affinity modeling, but that successful transfer remains strongly dependent on antibody-specific coverage and the limited size of the SARS-CoV-1 training set.

Under the dual-encoder neural-network architecture, KP1C9 was withheld during training and used as an independent validation antibody. This model showed a modest improvement in correlation (S1.1b_NN; Pearson’s r = 0.55, R² = 0.14, RMSE = 1.03), although predictive accuracy remained constrained by the limited size of the SARS-CoV-1 training dataset. These results suggest that representations learned from SARS-CoV-2 antibody:RBD interactions can transfer to related Sarbecoviruses and help prioritize experimentally testable SARS-CoV-1 antibody:variant pairs. However, modest absolute performance also underscores the difficulty of extrapolating across related viruses when labeled target-specific data remains sparse.

### 2.3 Similarity-guided experimental prioritization improves model performance through iterative dataset expansion

We refer to this step as heuristic model-guided expansion rather than formal active learning, because candidate selection was guided by model-derived feature-space coverage and sequence similarity rather than by a formal uncertainty-based acquisition function. This strategy was designed to be experimentally practical in a low-data setting, where calibrated uncertainty estimates may be unreliable.

To address the relatively weak performance of the initial SARS-CoV-1 predictor compared with its SARS-CoV-2 counterpart, we implemented a similarity-guided, model-informed dataset expansion strategy to expand both training datasets in a targeted, information-rich manner. Starting from datasets D1.1 and D2.1, we added new variant:HCAb interaction measurements, AL1.1 for SARS-CoV-1 and AL2.1 for SARS-CoV-2, chosen specifically to fill sparsely sampled regions of sequence and feature space. The expanded datasets were defined as D2.2 = D2.1 ∪ AL2.1 and D1.2 = D1.1 ∪ AL1.1. Rather than increasing dataset size indiscriminately, this strategy prioritized measurements expected to improve coverage of underrepresented antibody and antigen representations and thereby strengthen model generalization.

To further diversify the antibody component of the transfer-learning set, we projected HCAb feature representations into PCA space and compared antibodies already included in previous datasets with remaining candidate antibodies. Antibodies present in the existing transfer-learning set occupied several distinct regions of feature space, but additional underrepresented regions remained. We therefore prioritized antibodies located outside or at the periphery of the regions already covered by the previously selected set, reasoning that these candidates would contribute complementary paratope-related feature diversity rather than redundant sampling. Guided by this analysis, we selected AP3F2, AP3B8, BP1B10, DP3C8, EP3C11, and KP2B10 for further testing (**Supplementary Figure 3**). This PCA-guided expansion broadened antibody feature-space coverage and strengthened the diversity available for transfer-learning-based model refinement. In parallel, we mapped SARS-CoV-2 variants in PCA and sequence-similarity space and selected those most like SARS-CoV-1, thereby generating “bridge variants” that exposed the model to SARS-CoV-1-like sequence patterns without requiring large-scale additional assays (**Supplementary Figure 4**). PCA and sequence-similarity analyses together showed that SARS-CoV-1 variants are localized closest to a subset of WT-like SARS-CoV-2 variants rather than to the major Omicron-like clusters. We therefore selected SARS-CoV-2 bridge variants from this neighboring region to introduce SARS-CoV-1-like sequence patterns into the training set in a targeted manner. This targeted expansion improved predictive performance in both viral systems. For SARS-CoV-2, the updated models achieved Pearson’s r = 0.75, R² = 0.53, and RMSE = 0.62 for S2.2_NN, and Pearson’s r = 0.67, R² = 0.09, and RMSE = 0.85 for S2.3_NN. Correspondingly, the SARS-CoV-1 models improved to Pearson’s r = 0.56, R² = 0.13, and RMSE = 1.02 for S1.2_NN, and Pearson’s r = 0.68, R² = 0.36, and RMSE = 0.67 for S1.3_NN, after incorporation of AL2.1 followed by AL1.1, respectively (**Table 1**).

To determine whether model performance was specifically associated with the similarity-guided composition of AL1.1 and AL2.1, we performed a retrospective *in silico* comparison using matched random, PCA-similar, PCA-dissimilar/diversity-based, and reshuffled subsets of the existing measurements (**Supplementary Table 4**). For the SARS-CoV-1 model S1.3_NN, PCA-dissimilar/diversity-based selection produced the highest mean Pearson’s r (0.55 ± 0.21) and R² (0.29 ± 0.21), compared with random selection (r = 0.46 ± 0.15; R² = 0.15 ± 0.16) and PCA-similar selection (r = 0.51 ± 0.12; R² = 0.21 ± 0.13). The reshuffled datasets achieved a comparable mean correlation (0.54 ± 0.20) and the lowest RMSE (0.71 ± 0.12). These results suggest that increasing antibody feature-space diversity may be more beneficial than similarity-based selection for SARS-CoV-1 transfer, although performance remained dependent on the withheld antibody and data partition.

**Figure 5:**
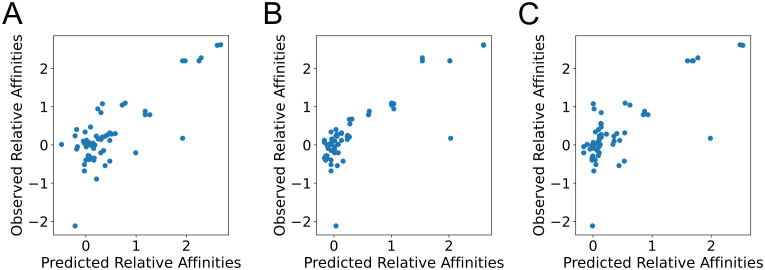
Combined withheld-antibody validation of the final SARS-CoV-1 model. Predicted versus observed relative affinities are shown for S1.4_NN when the HCAb KP1C9 was withheld together with one full-length conventional SARS-CoV-1 neutralizing antibody and used only for evaluation: **(A)** KP1C9 + CR3006, **(B)** KP1C9 + CR3013, and **(C)** KP1C9 + CR3014. Each point represents one SARS-CoV-1 RBD variant–antibody pair. The model recovered broad affinity trends across these representative combined holdouts, with the strongest performance observed for KP1C9 + CR3014. Expanded combined withheld antibody results across additional HCAb/full-length antibody combinations are provided in **Supplementary Table 2**. These results support use of the model for antibody:variant prioritization rather than fully resolved quantitative affinity prediction.

Finally, we incorporated full-length conventional anti–SARS-CoV-1 neutralizing antibodies (CR3006, CR3013, and CR3014) into the model-guided expanded datasets built from AL1.1 and AL2.1, together with newly selected SARS-CoV-2 “bridge variants” and SARS-CoV-1 variants. This dataset expansion improved performance in both viral systems, although gains remained antibody dependent. For SARS-CoV-2, the final model S2.4_NN achieved a mean Pearson correlation of 0.71 ± 0.12 across combined HCAb/full-length conventional antibody holdouts, with the representative HCAb KP1C9 + full-length conventional antibody holdouts shown in **Supplementary Figure 5**. In this representative SARS-CoV-2 analysis, Pearson correlations ranged from 0.75 to 0.84. For SARS-CoV-1, the final model S1.4_NN achieved a mean Pearson correlation of 0.63 ± 0.09 across combined withheld-antibody evaluations (**Supplementary Table 2**). **Figure 5** shows the corresponding representative SARS-CoV-1 combined holdout analysis in which KP1C9 was withheld together with CR3006, CR3013, or CR3014; the strongest performance in this set was observed for KP1C9 + CR3014 (Pearson’s r = 0.78, R² = 0.57, RMSE = 0.56). These results suggest that integrating conventional antibodies with targeted variant enrichment improves antibody:variant prioritization across closely related Sarbecoviruses but does not eliminate antibody-dependent variation in predictive accuracy.

## 3 Discussion

We developed an experimental and ML pipeline to predict antibody binding across diverse SARS-CoV RBD variants using limited data. Using epitope-focused antigen features and CDR descriptors in a dual-encoder neural-network architecture, the model emphasized interaction-relevant signals while reducing dependence on unconstrained global sequence representations (12, 13). Rather than relying on very large training compendia, the approach recovered broad affinity trends across several withheld-antibody evaluations under limited-data conditions, supporting its use as a practical tool for prioritizing antibody responses to viral variation.

A major strength of the framework is that it integrates both HCAbs aventional antibodies within a unified modeling strategy. This allowed us to move beyond a narrowly optimized within-dataset predictor toward a reusable representation of antibody recognition that could be iteratively expanded as new sequence and binding data became available. Coupled with similarity-guided, model-informed data expansion, the framework identifies sparsely sampled regions of antigen:antibody feature space where additional measurements are expected to be informative. Model-guided experimentation therefore becomes a practical mechanism for improving predictive performance while minimizing experimental burden, an important consideration for rapid-response studies of emerging viral threats.

Furthermore, focusing on conserved, experimentally grounded epitope patches provided a biologically interpretable representation that was especially useful for cross-virus transfer. We view the main value of epitope restriction here as providing a biologically grounded and transferable encoding that aligns homologous functional regions across related viral RBDs (14). This representation also reduces input complexity, facilitates comparison across viruses with partially conserved binding surfaces, and constrains the model to features most directly linked to antibody recognition.

More broadly, the interpretable feature design serves as a form of biological regularization (6, 15). By limiting antigen inputs to aligned epitope patches and antibody inputs to CDR-derived descriptors, the model is encouraged to make predictions from homologous, experimentally interpretable interaction determinants rather than from unconstrained global sequence correlations. This feature-constrained design is particularly useful in low-data transfer learning, where highly expressive models may otherwise learn source-domain patterns that do not transfer reliably to the target virus. The predicted-versus-observed plots also showed compression of predicted values for subsets of withheld antibodies, visible as vertical banding in several LOAO evaluations. This pattern is consistent with regression-to-the-mean behavior and likely reflects the combined effects of limited training-set diversity, mean-squared-error optimization, and the use of compact hand-crafted epitope-patch and CDR-derived descriptors. We intentionally used these descriptors rather than larger protein-language-model or geometric representations because the SARS-CoV-1 dataset was small and because homologous, interpretable features were needed for cross-virus alignment. However, this design likely limits model expressiveness. Thus, the model should be interpreted as a coarse prioritization tool that captures broad affinity trends rather than as a fully resolved quantitative affinity regressor.

Baseline comparisons clarify both the strengths and limitations of the neural-network framework in the SARS-CoV-2 source-virus model and the SARS-CoV-1 target-virus model. For the SARS-CoV-2 paired withheld-antibody evaluations, S2.4_NN provided the most balanced performance across Pearson correlation, R², and RMSE, with Pearson’s r = 0.71 ± 0.12, R² = 0.42 ± 0.19, and RMSE = 0.73 ± 0.13. In comparison, S2.4_LR showed similar average ranking performance but poorer quantitative accuracy, with Pearson’s r = 0.68 ± 0.12, R² = -0.03 ± 0.39 and RMSE = 0.96 ± 0.22, while S2.4_k-NN achieved Pearson’s r = 0.66 ± 0.14, R² = -0.357 ± 1.05 and RMSE = 1.02 ± 0.37. These results indicate that simple models captured some binding trends, but the neural-network model provided better overall accuracy for the SARS-CoV-2 source task. For the SARS-CoV-1 target, we also evaluated linear regression and k-nearest-neighbor regression using the same data partitioning used for S1.4 model evaluation (**Supplementary Table 3**). The S1.4_LR baseline achieved Pearson’s r = 0.68, R² = 0.44, and RMSE = 0.63, indicating that a simple linear model remains competitive on this smaller target-virus dataset. In contrast, S1.4_k-NN performed poorly, with Pearson’s r = 0.25, R² = −0.02, and RMSE = 0.86, suggesting that local similarity-based interpolation does not generalize well under antibody withholding. The S1.4_NN model achieved moderate performance across paired withheld-antibody evaluations, with Pearson’s r = 0.63 ± 0.09, R² = 0.35 ± 0.14, and RMSE = 0.68 ± 0.08. Thus, S1.4_NN was substantially better than k-NN and broadly comparable to LR, although it did not numerically exceed the LR baseline in this aggregate S1.4 comparison. We therefore interpret the benefit of the neural-network model not as uniform superiority over simpler baselines on every small dataset, but as its ability to support transfer learning from SARS-CoV-2 to SARS-CoV-1, staged fine-tuning, layer freezing, reusable antibody–antigen representations, and iterative expansion as additional binding data become available. Future work should benchmark these compact descriptors against antibody language-model embeddings, protein language-model antigen embeddings, and structure-aware antibody:antigen interface models under the same LOAO and cluster-based holdout conditions.

A notable advantage of the framework is its modularity. New epitope definitions, antigen encoders, or antibody feature representations can be incorporated without redesigning the workflow. This flexibility is especially useful in outbreak settings, where available information is often uneven and datasets evolve rapidly. Key limitations remain, including the current reliance on heuristic active-learning rules rather than explicit uncertainty estimation, and the need for broader validation across more diverse viral families to define the limits of cross-virus transferability. Future versions could also incorporate newer protein language model–based epitope or interface predictors and more explicit uncertainty-aware acquisition strategies (16). Importantly, the present results demonstrate feature-constrained transfer within a related viral subgenus rather than universal generalization to phylogenetically distant pathogens. Future work will also have to validate the expansion of this transfer learning approach beyond closely related antigens within a subgenus to the entire genus including phylogenetically distant members such as MERS-CoV and divergent bat coronaviruses. Epitope mapping across conserved antigen regions and independent model validation will determine whether predictive performance is retained across larger evolutionary distance.

Overall, this study provides a practical framework for prioritizing antibody binding in data-limited settings by combining targeted experiments with transferable, functionally aligned machine-learning representations. Transfer learning from SARS-CoV-2 supported useful prediction of SARS-CoV-1 antibody:variant interactions despite limited target-virus data, and iterative dataset expansion improved performance for several withheld-antibody evaluations. However, because SARS-CoV-1 and the selected SARS-CoV-2 variants occupy related regions of the engineered epitope-feature space, these results are best interpreted as local transfer across closely related Sarbecoviruses rather than broad *de novo* prediction across distant viral antigens. The key advance is therefore not simply neural-network architecture, but a data-efficient transfer-learning strategy in which interpretable epitope and CDR features constrain model behavior, guide targeted experiments, and enable local transfer from a data-rich Sarbecovirus to a related data-limited virus. These findings support an emerging-pathogen response framework in which functionally aligned representations from data-rich viruses are adapted through targeted measurements to guide experimental prioritization.

## 4 Methods

### 4.1 Machine learning modeling

#### 4.1.1 Data sources and processing

We assembled a multi-source dataset combining public, in-house, and computationally derived information to support epitope-focused modeling and cross-species transfer learning. Publicly available binding data describing interactions between diverse HCAbs and SARS-CoV-2 variants were obtained from our previously published studies (9) and related published analyses. These public datasets were integrated with our in-house SARS-CoV-2 antibody-binding measurements generated through iterative model-guided experimental expansion.

To extend the sequence space and enable transfer learning to SARS-CoV-1, we additionally incorporated experimentally measured in-house relative affinity values for *in silico*-generated SARS-CoV-1 RBD variant sequences. Cross-reactivity with conventional antibodies was evaluated by including binding data for three well-characterized SARS-CoV-1 neutralizing antibodies, CR3006, CR3013, and CR3014 (17).

The overall workflow consisted of: (i) defining functional epitope regions within the SARS-CoV-2 RBD; (ii) transferring those annotations to SARS-CoV-1; (iii) engineering antigen and antibody features restricted to functional binding regions; (iv) training neural-network models on SARS-CoV-2 and SARS-CoV-1 relative affinity datasets; and (v) refining model performance through similarity-guided experimental expansion and validation.

#### 4.1.2 Site-level functional scoring and epitope definition

To quantify functional importance across the RBD, we computed site-level scores by integrating public deep mutational scanning (DMS) data with variant- and structure-informed modifiers. This epitope definition was established *a priori* to generate biologically interpretable antigen features for transfer-learning model inputs, rather than to optimize model performance across alternative epitope definitions. Single-amino-acid mutation effects on ACE2 binding and RBD expression from Starr et al. (2020), scaled to a range of -2 to 5, served as the primary functional signal. These baseline values were augmented by adding +1 to sites carrying Omicron BA.1 mutations, +1 to sites carrying BA.4/5 mutations, and +1 to positions directly contacting ACE2 in available structures, yielding final per-site functional scores. These modifiers were included to prioritize RBD positions with functional, variant-associated, or structural evidence of relevance to receptor engagement and antibody recognition.

To identify higher-order epitope patches, site scores were aggregated across contiguous residue windows and ranked. Regions were iteratively selected in descending order of aggregate score until no window exceeded a score threshold of 1, producing a prioritized set of candidate functional patches. To evaluate potential cooperative effects between regions, we applied an ACE2 epistasis model to compute residuals for each variant and regressed these residuals on co-mutation counts across seven structurally defined RBD regions, corresponding to 28 possible region pairs. Region pairs exhibiting statistically significant associations (t ≥ 2) were designated as putative epistatic interactions, highlighting spatially or functionally coupled patches for downstream modeling and mutational design.

To refine epitope boundaries, we incorporated complementary conformational and structural information. The final epitope annotation was defined as the union of residues identified across the candidate functional patches, epistatic regions, conformational epitope predictions, and interaction-based structural epitope mapping. Adjacent epitope patches were then merged when separated by fewer than two amino acids, yielding ten spatially coherent functional motifs. To transfer this annotation to SARS-CoV-1, the SARS-CoV-1 and SARS-CoV-2 RBD sequences were aligned, and the corresponding epitope positions were mapped between the two viruses.

#### 4.1.3 Antibody feature engineering

Feature engineering was performed to capture both antigen- and antibody-specific determinants of binding while maintaining compatibility across SARS-CoV-2 and SARS-CoV-1 datasets. For antigen representation, only residues within the selected epitope patches were used. Sequence encoding of each epitope patch was performed using the extractBLOSUM function in the protR library with arguments submat = “AABLOSUM62”, k = 2, lag = 3, and scale = TRUE. This yielded 12 numerical features per epitope patch and 120 total numerical features per viral variant.

For antibody representation, only residues corresponding to the complementarity-determining regions (CDRs) of the heavy chain were used prior to encoding. For both conventional antibodies and HCAbs, CDR sequences were encoded using two complementary descriptor sets from the protR library: extractDescScales with propmat = “AATopo”, index = c(37:41, 43:47), pc = 2, and lag = 2; and extractSOCN with nlag = 4. These encodings yielded eight numerical features per CDR and 24 total numerical features per antibody sequence. For conventional antibodies, light-chain sequences were not included. To distinguish HCAb : SARS-CoV and conventional antibody:SARS-CoV interactions, an additional one-hot encoded feature indicating antibody class was included. All feature matrices were normalized with z-score. Model performance was evaluated using two validation paradigms: an 80/20 random split for baseline performance assessment and a leave-one-antibody-out (LOAO) strategy to quantify generalization to unseen antibodies.

#### 4.1.4 Determination of dissociation constant and relative affinity target variable

For every protein: antibody pair, multiple binding values B, were measured for varying concentrations x of antibodies. Using maximum-likelihood estimation, binding dissociation constants were determined by fitting the measured values to the Michaelis-Menten (MM) equation with background and log-scaled concentrations:


$$B=\;\frac{B_{max}\;}{1+\;10^{K_{d}-x}}+b_{g},$$

where $B_{max}$ is the maximum measured value for the antibody:protein pair, $K_{d}$ is the dissociation constant defined when $B=\frac{1}{2}B_{max}$, x is the concentration in units of log_10_(µg/mL), and $b_{g}$ is the measurement background. The likelihood L to be minimized is of the form


$$\ln(L)=\sum_{i}\ln(t_{\frac{B_{i}-{\hat{B}}_{i}(\theta )}{\sigma },4})-\ln(\sigma ),$$

where $t_{\alpha ,n}$ is the t-distribution with n degrees of freedom evaluated at α, $B_{i}$ is the observed binding value of the ith measurement, ${\hat{B}}_{i}(\theta )$ is the fitted binding value of the ith measurement for parameter set θ, and σ is a fitted error term. A t-distribution of four degrees of freedom was used to increase robustness and to account for the long tails of the experimental data. Optimization was performed using the Nelder-Mead algorithm with the following constraints: $0\leq B_{max}\leq 2\times \text{max}B)$, $\min(x)-1\leq K_{d}\leq \max(x)+2$, and $0\leq b_{g}\leq 1$.

A constant background model, $B=b_{g}\;$was also fitted and the Akaike Information Criterion (AIC) was computed for each model. The model with the lowest AIC was chosen to be the best, and protein: antibody pairs where the constant model was chosen were discarded from further modeling.

For the antibody:SARS-CoV variant interactions, we defined a relative affinity value $a_{\text{ab},\text{var}}^{R}$ defined as the $\text{log}(K_{d}^{ab:var})-\text{log}(K_{d}^{ab:WT})$, where $K_{d}^{ab:var}$ was defined to be the dissociation constant between a particular SARS-CoV variant and antibody, and $K_{d}^{\text{ab}:\text{WT}}$ was defined to be the between the SARS-CoV-2 WT variant and the same antibody. All $a_{ab,var}^{R}$ values were used as target variables for downstream predictions.

#### 4.1.5 Neural network architectures and training

To predict the relative affinity of each SARS-CoV–antibody pair, we trained both standard fully connected neural networks and dual-encoder neural-network models. For models using the dual-encoder architecture, antibody and antigen features were processed through separate fully connected encoder branches. The antibody branch encoded CDR-derived descriptors, whereas the antigen branch encoded SARS-CoV epitope-patch or variant-derived features. Each branch consisted of two fully connected layers.

For models trained on datasets containing both HCAbs and conventional full-length conventional antibodies, the latent representations from the antibody and antigen branches were concatenated with a one-hot-encoded antibody-class indicator representing HCAb versus conventional antibody. The concatenated representation was then passed through downstream fully connected layers to predict relative affinity. Models trained in part on the model-guided expansion datasets AL1.1 and AL2.1 used one additional fully connected layer after concatenation, whereas the remaining dual-encoder models used two additional fully connected layers.

The final output layer consisted of a single neuron that predicted the relative affinity value for each SARS-CoV antigen:antibody pair. For models not using the dual-encoder architecture, all input features were passed through a standard three-layer fully connected neural network, with a single-neuron output layer for relative-affinity prediction. Across all models, hidden layers used rectified linear unit (ReLU) activation, whereas the final output neuron used no activation function.

All non-categorical features in the training set were normalized using z-score scaling, and test-set features were scaled using the mean and standard deviation calculated from the corresponding training set. Models were trained using the Adam optimizer with default parameters and a mean-squared error loss function. Training was run for a maximum of 1000 epochs, with early stopping applied when test-set loss did not decrease for 100 consecutive epochs. Models trained on relative SARS-CoV-2: antibody affinity data (datasets with the prefix “D2.”) are denoted with the prefix “S2.”, whereas models trained on relative SARS-CoV-1:antibody affinity data (datasets with the prefix “D1.”) are denoted with the prefix “S1.” (**Table 1**). To enable transfer learning between related Sarbecoviruses, parameters of all layers except the penultimate layer were held fixed between paired S2.X and S1.X models during backpropagation. Different combinations of neuron counts, and feature sizes were evaluated, and only the model with the best performance on the testing set was used for downstream validation (**Table 1**). Architectural hyperparameters corresponding to the reported models are provided in **Supplementary Table 1**.

#### 4.1.6 K-nearest neighbor and elastic net regression model

To determine if model complexity was needed, using the same data partitioning, featurization, and scaling methods as the neural network models trained on the most number of SARS-CoV-2:antibody and SARS-CoV-1:antibody pairs and that produced the best performance metrics, we trained both an k-nearest-neighbor regression model as well as an elastic net regression model, which is a linear regression model with combined Ridge and Lasso coefficient penalties. For the KNN model, using the Euclidean distance as the distance metric, the value k defined as number of neighboring data points over which an average was computed to predict a new data point, was selected from the set {2,3}∪{k |5≤k≤100, k mod 5=0}. For the elastic net model, the Ridge and Lasso coefficient penalty values were both selected in the range [0,1] with incrementing values of 0.1. For both the KNN and the elastic net regression models, only the models containing the hyperparameters with the best performance on the testing set were reported.

#### 4.1.7 Similarity analysis and model-guided experimental expansion

To guide experimental prioritization and refine cross-species predictive performance, we combined feature-based and sequence-based similarity analyses within a heuristic model-guided expansion framework. First, protR-derived epitope features were reduced to two principal components using PCA to visualize and quantify similarity relationships across all SARS-CoV-2 and SARS-CoV-1 variants. The feature-based similarity was determined by first using the z-score scaled SARS-CoV-2 and SARS-CoV-1 epitope features from protR (Task 1 and reducing the dimensionality of the features to two dimensions via principal component analysis (PCA). Clusters of SARS-CoV-2 sequences that were closest in Euclidean distance to the clusters of SARS-CoV-1 sequences in PC space were ultimately denoted as the set of sequences as determined via feature-based similarity. The alignment-based similarity was determined by first filtering out sequences that contained deletion mutations. For each pair of sequences, X  and Y , with residues $X_{i}$ and $Y_{i}$ at position i  in the set of epitopes positions E , a normalized alignment score S(X,Y) between sequences X and Y was determined via the following equation:


$$S(X,Y)=\frac{2\sum_{i\in E}^{M}[X_{i},Y_{i}]}{\sum_{i\in E}^{M}[X_{i},X_{i}]+\sum_{i\in E}^{M}[Y_{i},Y_{i}]}$$

where M[A,B] denotes the BLOSUM62 substitution values between amino acids A  and B . SARS-CoV-2 sequences $X^{\text{SARS}-\text{CoV}-2}$, with the largest values of $S(X^{\text{SARS}-\text{CoV}-2},Y^{\text{SARS}-\text{CoV}-1})$ were ultimately denoted as the set of sequences as determined via alignment-based similarity. Variants appearing within the intersection of feature-based and alignment-based similarity sets were selected for the next round of experimental testing. Newly measured SARS-CoV-1:antibody and SARS-CoV-2:antibody relative affinities were then incorporated into the training datasets, and models were retrained after each experimental batch.

To compare the similarity-guided expansion, we performed *in-silico* random, diversity-based, and addition similarity-based expansion by selecting 70 of the 138 HCAb pairs in the combined AL1.1 and AL2.1 datasets for model performance. All subsampling methods sampled at least 20 of the 35 HCAb:variant pairs in AL2.1 such that the subsample contained at least two unique HCAb sequences, and 50 of the 103 HCAb:variant pairs in AL1.1, such that the subsample contained at least three unique HCAb sequences. We tested our results on model S1.3_NN and S2.3_NN, which used LOAO validation. We defined diversity-based (similarity-based) selection by selecting HCAbs from the combined AL1.1 and AL2.1 that had the largest (smallest) Euclidean distances from the withheld HCAb.

### 4.2 Experimental validation

#### 4.2.1 Protein production of SARS-CoV-2 and SARS-CoV-1 variant RBDs

For antigen design, the SARS-CoV-1 spike protein was truncated to the RBD (residues 319–510), engineered with a C-terminal His tag and AviTag for biotinylation, and cloned into a plasmid backbone originally named pCMV. For SARS-CoV-2, the wild-type (Wuhan strain, GenBank: QHR63250.2) RBD construct (residues 331–524/531) was previously generated (PMID: 39847604) in the same backbone. All DNA fragments encoding SARS-CoV2 RBD variants were synthesized and cloned by Twist Bioscience Inc. following our specifications.

Viral antigen plasmids were expanded in-house using high-throughput transformations with Mix & Go! Competent DH5α 96 E. coli cells (Zymo Research), followed by purification with QIAGEN Plasmid Plus 96 Miniprep kits. Purified plasmids were used to transfect Expi293F cells (Thermo Fisher, A14527) in 5 mL cultures at a final concentration of 1 µg DNA/mL (4.5 µg SARS-CoV-1 or SARS-CoV-2 S plasmid plus 0.5 µg BirA plasmid for *in situ* biotinylation). At 18–22 hours post-transfection, ExpiFectamine™ 293 Transfection Enhancer 1 and Enhancer 2 were added according to the manufacturer’s instructions. Proteins were harvested 5–7 days post-transfection by centrifuging 24-well deep-well plates at 3000 × g for 10 minutes at 4°C to pellet cells. Supernatants were filtered through a 0.2 µm syringe filter and concentrated on ice using 3 kDa Amicon ultra centrifugal filters. Concentration was performed at 3000 × g for 1 hour at 4°C to reduce volumes to ~500 µL–1 mL. Proteins were then buffer exchanged by adding 9.5 mL PBS (pH 6.5) to the filter unit and centrifuging again under the same conditions. Final protein concentrations were determined by UV absorbance using a Nanodrop spectrophotometer.

#### 4.2.2 Conventional antibody and HCAb production

Seventeen HCAbs from our previous study (PMID: 39847604) AP1C3, KP1C9, BP3D5, BP2A6, AP1B6, BP1C8, AP1B1, BP1B1, DP4F2, AP1A5, BP2H9, DP3C8, KP2B10, EP3C11, AP3A2, AP3B8, and BP1B10 were produced from plasmids generated in that work.

Conventional anti-SARS-CoV-1 antibodies (CR3006, CR3013, and CR3014) were identified from the literature and cloned in-house using IDT gBlocks to assemble separate heavy-chain (HC) and light-chain (LC) expression constructs. Transfection-grade HC and LC plasmids were expanded using Mix & Go! Competent DH5α 96 E. coli cells (Zymo Research) and purified with QIAGEN Plasmid Plus MidiPrep kits. Conventional antibodies were expressed in ExpiCHO cells. Cultures (100 mL) were grown to the manufacturer-recommended density and transfected with HC and LC plasmids at a 1:1 ratio (50µg HC + 50µg LC) using Opti-PRO medium and ExpiFectamine CHO reagents. Post-transfection enhancers were added following the Max Titer protocol. Cultures were harvested on day 8 by centrifugation at 3000 × g for 10 minutes at 4°C. Supernatants were clarified through 0.2µm filtration and purified via Protein A affinity chromatography using a MabSelect PrismA column on an ÄKTA Pure FPLC system. Eluted antibodies were immediately neutralized with Tris buffer (pH 9), and fractions containing the IgG peak were further polished by size-exclusion chromatography (SEC) on a Superdex 200 Increase 10/300 GL column using 1× PBS as the mobile phase.

#### 4.2.3 Enzyme-linked immunosorbent assays

Purified SARS-CoV-1 and SARS-CoV-2 antigens and antibodies were evaluated using titration ELISAs against a panel of neutralizing antibodies, including three full-length conventional SARS-CoV-1/2 antibodies (CR3006, CR3013, CR3014) and seventeen HCAbs from PMID: 39847604 (AP1C3, KP1C9, BP3D5, BP2A6, AP1B6, BP1C8, AP1B1, BP1B1, DP4F2, AP1A5, BP2H9, DP3C8, KP2B10, EP3C11, AP3A2, AP3B8, and BP1B10). Streptavidin-coated 384-well plates were prepared by coating each well with SARS-CoV spike protein variants at 2 µg/mL in carbonate buffer (100 mM NaHCO_3_, 150 mM NaCl, pH 8.3) for 1 hour at room temperature on an orbital shaker (400 rpm). Plates were washed six times with wash buffer (1× PBS + 0.05% Tween-20) using a BioTek EL406 plate washer; this wash step was repeated after each subsequent incubation.

Wells were blocked with 50 µL/well Pierce Protein-Free Blocking Buffer (Thermo Fisher) for 2 hours at room temperature with shaking (400 rpm). After washing, primary antibodies were added in an eight-point titration series consisting of 5 µg/mL, 0.5 µg/mL, and six additional 1:3 serial dilutions down to 0.002058 µg/mL, plus a 0 µg/mL no-antibody control. All concentrations were tested in triplicate and incubated for 2 hours at room temperature with orbital shaking (400 rpm). Plates were washed and incubated with HRP-conjugated goat anti-human IgG secondary antibody (Thermo Fisher) diluted 1:15,000 for 1 hour under the same shaking conditions. Following a final wash, 25µL/well of TMB substrate was added for color development, and reactions were quenched after 5 minutes by adding 25µL/well of 2 M H_2_SO_4_. Absorbance at 450 nm was measured on a Tecan Spark Cyto plate reader. Binding titration curves were fit to determine the dissociation constant ( $K_{d}$) for each antigen:antibody pair.

## Data Availability

The datasets presented in this study can be found in online repositories. The names of the repository/repositories and accession number(s) can be found in the article/**Supplementary Material**.
