# Supplementary material for: Modeling antibody recognition across SARS-CoV-2 and SARS-CoV-1 using epitope-informed transfer learning
Source: Front Immunol. 2026 Aug 5;17:1886934. doi: 10.3389/fimmu.2026.1886934 (PMC13485751; doi:10.3389/fimmu.2026.1886934)
Supplement: Supplementary file 1 [file Table1.docx]

**SUPPLEMENTARY INFORMATION**

**Modeling antibody recognition across SARS-CoV-2 and SARS-CoV-1 using epitope-informed transfer learning**

Jimmy B. Yuan^1†^, Ryan C. Bruneau^1†^, Stephen Won^1^, Aidan Elliot Heller^2^, Thomas Sheffield^3^, Kenneth L. Sale^3^, Brooke Harmon^1^, Le Thanh Mai Pham^2*^

^1^Biotechnology and Bioengineering, Sandia National Laboratories, Livermore, California, United States of America

^2^Bioresource and Environmental Security, Sandia National Laboratories, Livermore, California, United States of America

^3^Biosecurity and Bioassurance, Sandia National Laboratories, Livermore, California, United States of America

^†^ Equal contributions

***** Corresponding authors:

Le Thanh Mai Pham, email: [lpham@sandia.gov](mailto:lpham@sandia.gov)

**Table S1.** **Neural-network architectures used for the S1 and S2 model series.** The table summarizes the number of neurons used in the antibody, variant, and combined modules for each model architecture. “N/A” indicates that the corresponding module was not used in that model.

| **Model** | **Antibody**  **Module Layer 1 Neurons** | **Antibody**  **Module Layer 2 Neurons** | **Variant**  **Module Layer 1**  **Neurons** | **Variant**  **Module Layer 2**  **Neurons** | **Combined**  **Module Layer 1**  **Neurons** | **Combined**  **Module Layer 2**  **Neurons** |
| --- | --- | --- | --- | --- | --- | --- |
| SX.1_NN | N/A | N/A | N/A | N/A | 5 | 10 |
| SX.1a_NN | N/A | N/A | N/A | N/A | 3 | 12 |
| SX.1b_NN | 4 | 2 | 6 | 8 | 8 | N/A |
| SX.2_NN | 4 | 2 | 6 | 6 | 10 | N/A |
| SX.3_NN | 6 | 3 | 6 | 2 | 8 | N/A |
| SX.4_NN | 6 | 3 | 6 | 2 | 10 | N/A |

**Table S2: Expanded withheld-antibody validation of final SARS-CoV-1 and SARS-CoV-2 models.**

LOAO performance is shown for final SARS-CoV-1 and SARS-CoV-2 models across combinations of withheld HCAbs and withheld full-length antibodies. Metrics include Pearson correlation, R², and RMSE. Summary rows report mean ± standard deviation across withheld-antibody evaluations. The results show that model performance is antibody-dependent, with stronger performance for some withheld antibody combinations and weaker performance for others, supporting use of the models for coarse antibody–variant prioritization rather than uniformly accurate quantitative affinity prediction.

| **Model** | **HCAb withheld** | **Full-length Ab withheld** | **Pearson’s r** | **R²** | **RMSE** |
| --- | --- | --- | --- | --- | --- |
| S1.4_NN | AP1B6 | CR3006 | 0.60 | 0.21 | 0.76 |
| S1.4_NN | AP1B6 | CR3013 | 0.59 | 0.35 | 0.69 |
| S1.4_NN | AP1B6 | CR3014 | 0.63 | 0.37 | 0.67 |
| S1.4_NN | AP1C3 | CR3006 | 0.67 | 0.41 | 0.65 |
| S1.4_NN | AP1C3 | CR3013 | 0.66 | 0.42 | 0.65 |
| S1.4_NN | AP1C3 | CR3014 | 0.73 | 0.52 | 0.59 |
| S1.4_NN | BP2A6 | CR3006 | 0.52 | 0.18 | 0.77 |
| S1.4_NN | BP2A6 | CR3013 | 0.55 | 0.17 | 0.77 |
| S1.4_NN | BP2A6 | CR3014 | 0.57 | 0.29 | 0.72 |
| S1.4_NN | BP3D5 | CR3006 | 0.49 | 0.12 | 0.79 |
| S1.4_NN | BP3D5 | CR3013 | 0.54 | 0.26 | 0.73 |
| S1.4_NN | BP3D5 | CR3014 | 0.71 | 0.50 | 0.59 |
| S1.4_NN | KP1C9 | CR3006 | 0.75 | 0.53 | 0.58 |
| S1.4_NN | KP1C9 | CR3013 | 0.67 | 0.42 | 0.64 |
| S1.4_NN | KP1C9 | CR3014 | 0.78 | 0.57 | 0.56 |
| **S1.4_NN mean ± SD** | — | — | **0.63 ± 0.09** | **0.35 ± 0.14** | **0.68 ± 0.08** |
| S2.4_NN | AP1B6 | CR3006 | 0.52 | 0.03 | 0.96 |
| S2.4_NN | AP1B6 | CR3013 | 0.53 | 0.13 | 0.93 |
| S2.4_NN | AP1B6 | CR3014 | 0.61 | 0.35 | 0.82 |
| S2.4_NN | AP1C3 | CR3006 | 0.78 | 0.61 | 0.56 |
| S2.4_NN | AP1C3 | CR3013 | 0.79 | 0.61 | 0.59 |
| S2.4_NN | AP1C3 | CR3014 | 0.80 | 0.64 | 0.59 |
| S2.4_NN | BP2A6 | CR3006 | 0.80 | 0.30 | 0.85 |
| S2.4_NN | BP2A6 | CR3013 | 0.85 | 0.44 | 0.82 |
| S2.4_NN | BP2A6 | CR3014 | 0.83 | 0.42 | 0.85 |
| S2.4_NN | BP3D5 | CR3006 | 0.57 | 0.27 | 0.68 |
| S2.4_NN | BP3D5 | CR3013 | 0.60 | 0.35 | 0.65 |
| S2.4_NN | BP3D5 | CR3014 | 0.60 | 0.36 | 0.66 |
| S2.4_NN | KP1C9 | CR3006 | 0.75 | 0.49 | 0.72 |
| S2.4_NN | KP1C9 | CR3013 | 0.84 | 0.68 | 0.58 |
| S2.4_NN | KP1C9 | CR3014 | 0.81 | 0.59 | 0.68 |
| **S2.4_NN mean ± SD** | — | — | **0.71 ± 0.12** | **0.42 ± 0.19** | **0.73 ± 0.13** |

Table S3. **Performance of S1.4 and S2.4 baseline regression models under paired antibody-withholding validation.** Linear regression (LR) and k-nearest-neighbor (k-NN) models were evaluated using Pearson correlation coefficient (r), coefficient of determination (R²), and root mean square error (RMSE). For S2.4 models, one heavy-chain-only antibody (HCAb) and one full-length conventional antibody were withheld from training in each evaluation to assess generalization to unseen antibody classes. The final rows report the mean ± standard deviation across all paired S2.4 withholding combinations.

| **Model** | **HCAb withheld** | **Conventional Ab withheld** | **Pearson’s r** | **R²** | **RMSE** |
| --- | --- | --- | --- | --- | --- |
| S1.4_LR |  |  | 0.68 | 0.44 | 0.63 |
| S2.4_LR | AP1B6 | CR3006 | 0.54 | -0.61 | 1.24 |
| S2.4_LR | AP1B6 | CR3013 | 0.53 | -0.40 | 1.18 |
| S2.4_LR | AP1B6 | CR3014 | 0.51 | -0.33 | 1.17 |
| S2.4_LR | AP1C3 | CR3006 | 0.73 | -0.34 | 1.03 |
| S2.4_LR | AP1C3 | CR3013 | 0.77 | 0.12 | 0.88 |
| S2.4_LR | AP1C3 | CR3014 | 0.76 | 0.17 | 0.88 |
| S2.4_LR | BP2A6 | CR3006 | 0.76 | -0.51 | 1.25 |
| S2.4_LR | BP2A6 | CR3013 | 0.79 | -0.26 | 1.21 |
| S2.4_LR | BP2A6 | CR3014 | 0.78 | -0.19 | 1.20 |
| S2.4_LR | BP3D5 | CR3006 | 0.50 | -0.07 | 0.82 |
| S2.4_LR | BP3D5 | CR3013 | 0.56 | 0.17 | 0.74 |
| S2.4_LR | BP3D5 | CR3014 | 0.55 | 0.13 | 0.77 |
| S2.4_LR | KP1C9 | CR3006 | 0.78 | 0.51 | 0.70 |
| S2.4_LR | KP1C9 | CR3013 | 0.80 | 0.55 | 0.69 |
| S2.4_LR | KP1C9 | CR3014 | 0.81 | 0.61 | 0.66 |
| **S2.4_LR mean ± SD** | **--** | **--** | **0.68 ± 0.12** | **-0.03 ± 0.39** | **0.96 ± 0.22** |
|  |  |  |  |  |  |
|  | **HCAb withheld** | **Conventional Ab withheld** | **Pearson’s r** | **R2** | **RMSE** |
| S1.4_k-NN |  |  | 0.25 | -0.02 | 0.86 |
| S2.4_k-NN | AP1B6 | CR3006 | 0.40 | 0.06 | 0.95 |
| S2.4_k-NN | AP1B6 | CR3013 | 0.43 | 0.09 | 0.95 |
| S2.4_k-NN | AP1B6 | CR3014 | 0.44 | 0.11 | 0.96 |
| S2.4_k-NN | AP1C3 | CR3006 | 0.75 | 0.44 | 0.67 |
| S2.4_k-NN | AP1C3 | CR3013 | 0.77 | 0.48 | 0.67 |
| S2.4_k-NN | AP1C3 | CR3014 | 0.78 | 0.52 | 0.67 |
| S2.4_k-NN | BP2A6 | CR3006 | 0.66 | -1.23 | 1.51 |
| S2.4_k-NN | BP2A6 | CR3013 | 0.71 | -0.95 | 1.50 |
| S2.4_k-NN | BP2A6 | CR3014 | 0.70 | -0.84 | 1.49 |
| S2.4_k-NN | BP3D5 | CR3006 | 0.55 | -2.34 | 1.45 |
| S2.4_k-NN | BP3D5 | CR3013 | 0.61 | -1.89 | 1.38 |
| S2.4_k-NN | BP3D5 | CR3014 | 0.63 | -1.79 | 1.37 |
| S2.4_k-NN | KP1C9 | CR3006 | 0.81 | 0.64 | 0.59 |
| S2.4_k-NN | KP1C9 | CR3013 | 0.83 | 0.67 | 0.59 |
| S2.4_k-NN | KP1C9 | CR3014 | 0.83 | 0.68 | 0.59 |
| **S2.4_k-NN mean ± SD** | **--** | **--** | **0.66 ± 0.14** | **-0.357 ± 1.05** | **1.02 ± 0.37** |

Table S4. **Retrospective comparison of random, PCA-similar, PCA-dissimilar/diversity-based, and reshuffled data-selection strategies for S2.3_NN and S1.3_NN under leave-one-antibody-out validation.**

| **Model** | **Condition** | **Split seed** | **HCAb withheld** | **Pearson’s r** | **R²** | **RMSE** |
| --- | --- | --- | --- | --- | --- | --- |
| S2.3_NN | Random |  | AP1B6 | 0.32 | 0.05 | 0.87 |
| S2.3_NN | Random |  | AP1C3 | 0.65 | 0.39 | 0.59 |
| S2.3_NN | Random |  | BP2A6 | 0.72 | 0.51 | 0.58 |
| S2.3_NN | Random |  | BP3D5 | 0.42 | 0.16 | 0.68 |
| S2.3_NN | Random |  | KP1C9 | 0.70 | 0.41 | 0.68 |
| **S2.3_NN mean ± SD** |  |  |  | **0.58 ± 0.18** | **0.30 ± 0.19** | **0.68 ± 0.12** |
| S2.3_NN | PCA-similar |  | AP1B6 | 0.36 | 0.07 | 0.86 |
| S2.3_NN | PCA-similar |  | AP1C3 | 0.68 | 0.44 | 0.56 |
| S2.3_NN | PCA-similar |  | BP2A6 | 0.71 | 0.33 | 0.68 |
| S2.3_NN | PCA-similar |  | BP3D5 | 0.37 | 0.06 | 0.72 |
| S2.3_NN | PCA-similar |  | KP1C9 | 0.73 | 0.47 | 0.65 |
| **S2.3_NN mean ± SD** |  |  |  | **0.57 ± 0.19** | **0.27 ± 0.20** | **0.69 ± 0.11** |
| S2.3_NN | PCA-dissimilar |  | AP1B6 | 0.35 | 0.03 | 0.88 |
| S2.3_NN | PCA-dissimilar |  | AP1C3 | 0.65 | 0.38 | 0.59 |
| S2.3_NN | PCA-dissimilar |  | BP2A6 | 0.66 | 0.42 | 0.63 |
| S2.3_NN | PCA-dissimilar |  | BP3D5 | 0.40 | 0.14 | 0.69 |
| S2.3_NN | PCA-dissimilar |  | KP1C9 | 0.68 | 0.31 | 0.74 |
| **S2.3_NN mean ± SD** |  |  |  | **0.55 ± 0.16** | **0.26 ± 0.17** | **0.71 ± 0.11** |
| S2.3_NN | 80/20 reshuffle | 42 | AP1B6 | 0.35 | 0.01 | 0.89 |
| S2.3_NN | 80/20 reshuffle | 42 | AP1C3 | 0.67 | 0.32 | 0.62 |
| S2.3_NN | 80/20 reshuffle | 42 | BP2A6 | 0.73 | 0.33 | 0.68 |
| S2.3_NN | 80/20 reshuffle | 42 | BP3D5 | 0.31 | 0.02 | 0.74 |
| S2.3_NN | 80/20 reshuffle | 42 | KP1C9 | 0.77 | 0.54 | 0.60 |
| S2.3_NN | 80/20 reshuffle | 888 | AP1B6 | 0.34 | -0.07 | 0.92 |
| S2.3_NN | 80/20 reshuffle | 888 | AP1C3 | 0.65 | 0.41 | 0.58 |
| S2.3_NN | 80/20 reshuffle | 888 | BP2A6 | 0.70 | -0.28 | 0.94 |
| S2.3_NN | 80/20 reshuffle | 888 | BP3D5 | 0.36 | 0.12 | 0.70 |
| S2.3_NN | 80/20 reshuffle | 888 | KP1C9 | 0.81 | 0.59 | 0.57 |
| **S2.3_NN mean ± SD** |  |  |  | **0.57 ± 0.20** | **0.20 ± 0.28** | **0.72 ± 0.14** |
| S1.3_NN | Random |  | AP1B6 | 0.27 | -0.06 | 1.10 |
| S1.3_NN | Random |  | AP1C3 | 0.63 | 0.33 | 0.87 |
| S1.3_NN | Random |  | BP2A6 | 0.52 | 0.25 | 0.93 |
| S1.3_NN | Random |  | BP3D5 | 0.35 | 0.03 | 1.05 |
| S1.3_NN | Random |  | KP1C9 | 0.54 | 0.22 | 0.94 |
| **S1.3_NN mean ± SD** |  |  |  | **0.46 ± 0.15** | **0.15 ± 0.16** | **0.98 ± 0.09** |
| S1.3_NN | PCA-similar |  | AP1B6 | 0.39 | 0.09 | 0.97 |
| S1.3_NN | PCA-similar |  | AP1C3 | 0.68 | 0.41 | 0.76 |
| S1.3_NN | PCA-similar |  | BP2A6 | 0.57 | 0.27 | 0.86 |
| S1.3_NN | PCA-similar |  | BP3D5 | 0.39 | 0.13 | 0.93 |
| S1.3_NN | PCA-similar |  | KP1C9 | 0.50 | 0.13 | 0.93 |
| **S1.3_NN mean ± SD** |  |  |  | **0.51 ± 0.12** | **0.21 ± 0.13** | **0.89 ± 0.08** |
| S1.3_NN | PCA-dissimilar |  | AP1B6 | 0.34 | 0.10 | 0.94 |
| S1.3_NN | PCA-dissimilar |  | AP1C3 | 0.69 | 0.43 | 0.76 |
| S1.3_NN | PCA-dissimilar |  | BP2A6 | 0.74 | 0.44 | 0.76 |
| S1.3_NN | PCA-dissimilar |  | BP3D5 | 0.31 | 0.04 | 0.99 |
| S1.3_NN | PCA-dissimilar |  | KP1C9 | 0.69 | 0.46 | 0.74 |
| **S1.3_NN mean ± SD** |  |  |  | **0.55 ± 0.21** | **0.29 ± 0.21** | **0.84 ± 0.12** |
| S1.3_NN | 80/20 reshuffle | 42 | AP1B6 | 0.27 | 0.07 | 0.86 |
| S1.3_NN | 80/20 reshuffle | 42 | AP1C3 | 0.68 | 0.45 | 0.66 |
| S1.3_NN | 80/20 reshuffle | 42 | BP2A6 | 0.72 | 0.50 | 0.63 |
| S1.3_NN | 80/20 reshuffle | 42 | BP3D5 | 0.41 | 0.15 | 0.82 |
| S1.3_NN | 80/20 reshuffle | 42 | KP1C9 | 0.62 | 0.38 | 0.70 |
| S1.3_NN | 80/20 reshuffle | 888 | AP1B6 | 0.32 | 0.03 | 0.80 |
| S1.3_NN | 80/20 reshuffle | 888 | AP1C3 | 0.61 | 0.34 | 0.66 |
| S1.3_NN | 80/20 reshuffle | 888 | BP2A6 | 0.63 | 0.37 | 0.65 |
| S1.3_NN | 80/20 reshuffle | 888 | BP3D5 | 0.29 | -0.03 | 0.83 |
| S1.3_NN | 80/20 reshuffle | 888 | KP1C9 | 0.82 | 0.65 | 0.48 |
| **S1.3_NN mean ± SD** |  |  |  | **0.54 ± 0.20** | **0.29 ± 0.22** | **0.71 ± 0.12** |

**Figure S1: SARS-CoV-2 variant coverage across antibodies used to prioritize candidates for LOAO analysis.** Bars show the number of SARS-CoV-2 RBD variants associated with each antibody in the dataset. Antibodies with the largest numbers of measured variant interactions, including BP2A6, BP3D5, and KP1C9, were prioritized for LOAO evaluation to ensure sufficiently large holdout sets for meaningful assessment of generalization.

**Figure S2:** **protR-derived CDR feature distributions for candidate LOAO antibodies in the SARS-CoV-2 dataset.** For each of the 24 CDR-derived features, histograms compare the distribution of values for the remaining antibodies in the training set with the feature value of the candidate held-out antibody. Top, BP3D5 compared with AP1B6 and AP1C3; bottom, BP2A6 compared with KP1C9. Across multiple features, KP1C9 showed broader overlap with the feature-space support of the remaining antibodies. In contrast, BP2A6 and BP3D5 more frequently occupied peripheral or sparsely represented regions, consistent with weaker LOAO generalization. This qualitative pattern is consistent with the stronger LOAO generalization observed for KP1C9.

**Figure S3: PCA-guided selection of additional antibodies for transfer-learning set diversification.** HCAbs that were highly represented in previous large datasets are highlighted in blue. To broaden paratope-related feature-space coverage and reduce redundancy, additional antibodies were selected from underrepresented or peripheral regions of PCA space. The newly prioritized candidates, highlighted in red, were BP1B10, AP3B8, DP3C8, KP2B10, AP3F2, and EP3C11.

**Figure S4. Similarity-based selection of SARS-CoV-2 bridge variants for model-guided experimental expansion.** (A) PCA of protR-derived epitope features for SARS-CoV-2 and SARS-CoV-1 variants. Blue points indicate SARS-CoV-2 variants and red points indicate SARS-CoV-1 variants. Candidate SARS-CoV-2 bridge variants were selected from regions of PCA space nearest to SARS-CoV-1 variants. (B) Heatmap of pairwise alignment scores between SARS-CoV-2 and SARS-CoV-1 variants containing only point mutations in epitope regions. Final SARS-CoV-2 bridge variants were selected using the intersection of feature-based similarity from PCA and sequence-based similarity from alignment scoring.

**Figure S5. Combined withheld-antibody validation of the final SARS-CoV-2 model.** Scatter plots show observed versus predicted relative affinities for S2.4_NN when the HCAb KP1C9 was withheld together with one full-length antibody and used only for evaluation: (A) KP1C9 + CR3006, (B) KP1C9 + CR3013, and (C) KP1C9 + CR3014. Each point represents one SARS-CoV-2 RBD variant–antibody pair. The model recovered broad affinity trends across these combined holdouts, with Pearson r = 0.75, R² = 0.49, RMSE = 0.72 for KP1C9 + CR3006; Pearson r = 0.84, R² = 0.68, RMSE = 0.58 for KP1C9 + CR3013; and Pearson r = 0.81, R² = 0.59, RMSE = 0.68 for KP1C9 + CR3014. Expanded combined withheld-antibody metrics are provided in Supplementary Table S2.
